# Febrile Proteinuria in Hospitalized Children: Characterization of Urinary Proteins

**DOI:** 10.3389/fped.2018.00202

**Published:** 2018-08-17

**Authors:** Evgenia Gurevich, Eytan Israel, Yael Segev, Daniel Landau

**Affiliations:** ^1^Soroka University Medical Center, Ben-Gurion University of the Negev, Beersheba, Israel; ^2^Division of Pediatrics, Ben-Gurion University of the Negev, Beersheba, Israel; ^3^Department of Microbiology and Immunology, Ben-Gurion University of the Negev, Beersheba, Israel; ^4^Department of Pediatrics B, Schneider Children's Medical Center, Sackler School of Medicine, Tel Aviv University, Tel Aviv, Israel

**Keywords:** β2-microglobulin, uromodulin, CD80 (B7-1) protein, albuminuria, proteinuria, fever

## Abstract

**Background:** Transient proteinuria during febrile illness is a common phenomenon. Recent studies have re-examined the pathophysiology of proteinuria and new urinary markers to characterize it, including B7-1 (CD80), which is expressed also in glomerular podocytes and influences the glomerular barrier.

**Aim:** To investigate the pattern of proteinuria in febrile non-renal diseases, including B7-1.

**Methods:** We prospectively analyzed urine samples of 44 febrile children and 28 afebrile controls for different protein components: albumin (glomerular marker), β2-microglobulin (tubular marker), uromodulin (Tamm Horsfall protein-THP, a renal endogenous protein) and B7-1. Febrile illness was characterized as focal bacterial vs. viral. Exclusion criteria were underlying renal disease, steroid treatment or urinary tract infection.

**Results:** Elevated urine albumin (64.5 ± 10.3 vs. 17.8 ± 4 mg/g, mean ± S.E.M., *p* = 0.0009) and β2-microglobulin (1.44 ± 0.34 vs. 0.182 ± 0.03 mg/g, mean ± S.E.M., *p* = 0.005] and decreased uromodulin (10.5 ± 1 vs. 26.7 ± 2.2 Arbitrary units, mean ± S.E.M., *p* = 0.0001) excretion were found during febrile illness vs. controls. Urine B7-1 was also increased in the febrile group (0.27 ± 0.05 vs. 0.07 ± 0.01 ng/ml, mean ± S.E.M., *p* = 0.001), and was the only marker which was significantly higher in bacterial vs. viral disease.

**Conclusions:** Febrile proteinuria is not generalized: while proteins of both glomerular and tubular origin increase, uromodulin decreases. Urine B7-1 is increased during fever, more significantly in bacterial infections. Thus, urinary B7-1 may be used as an additional marker to differentiate between febrile states of bacterial vs. viral origin.

## Introduction

Normal glomerular barrier prevents the filtration of high molecular weight proteins into the Bowman's capsule. Subsequently, proximal tubular reabsorption retrieves proteins of lower molecular weight that escaped glomerular barrier, leading to a minimal amount of protein of circulation origin in urine. In addition, uromodulin, or Tamm Horsfall protein is synthesized and partly secreted by the distal tubule and found in urine in normal states (1, 2). Transient proteinuria is a common finding in several illness states. It has been described during fever, sepsis, trauma and anaphylaxis, without relation to renal disease (3). Several studies in the past have described transient proteinuria during febrile illness and whether its origin was glomerular or tubular (4, 5). However, no studies have been performed on the role of urinary uromodulin in this process. Recent studies have reported new evidence on the pathophysiology of infection related proteinuria, including the role of B7-1(CD80) protein, expressed on both circulating leucocytes as well as on glomerular epithelial cells (podocytes). This protein was shown to modulate the podocyte cytoskeleton and influence on glomerular filtration barrier in different proteinuric states (6, 7). No studies have been performed to simultaneously examine the involvement of markers of different renal compartments (glomerular, tubular, and endogenously synthesized), including B7-1, in this common phenomenon. We hypothesized that glomerular B7-1 may be upregulated also in transient febrile proteinuria. Therefore, in this study we characterized the type of urinary proteins (albumin, β2-microglobulin, uromodulin, and B7-1) in children with febrile proteinuria.

## Patients and methods

The research protocol of the study has been approved by the local Helsinki committee. Study consent was signed by patients' parents. In this prospective study, children with febrile illness (temperature above 38°C) of infectious origin, hospitalized in pediatric wards were enrolled. The exclusion criteria were underlying renal disease, signs of urinary tract infection, or corticosteroid treatment. Children without fever who were hospitalized for elective surgical procedures served as controls. Clinical, laboratory and imaging data were recorded for each patient and the child was finally diagnosed as having a viral [no specific focus of infection, normal peripheral blood leucocyte (WBC) count] or a specific bacterial disease.

Urine samples in both study and control groups were collected at the time of admission. Urine samples were checked with Multistix® urinalysis strips for white blood cells, nitrites, protein, blood and specific gravity. Then urine samples were biochemically tested for total protein, albumin (as glomerular injury marker), β2-microglobulin (tubular marker), uromodulin (Tamm Horsfall protein-THP, a renal endogenous protein), and B7-1 (CD-80) protein. Urine protein response on the sticks was determined using a semi quantitative determination (+1 to +4) method by photometric color test. Urine creatinine was determined using kinetic color test (Jaffé method). Urine albumin was determined by immune turbidometric test. All these tests were performed using an Olympus Life &[[Inline Image]]Material Science system. Urinary β2-microglobulin was determined using Microparticle Enzyme Immunoassay (MEIA)(Abbott Ax SYM, Germany). Tamm Horsfall protein (THP)/uromodulin concentration was analyzed by Western blot analysis using rabbit anti THP antibody (Santa Cruz). Protein expression was quantified densitometrically using *Image J* software and expressed as arbitrary units (AU). Urine B7-1 (CD80) protein concentration was analyzed using an enzyme-linked immunosorbent assay kit (Human sCD80 Instant Elisa Kit, eBioscience, Affymetrix, North America), according to the test protocol. Briefly, after a 3 h incubation of 50 μl urine samples at room temperature, the microwell strips were washed 3 times with approximately 400 μl wash buffer per well. Then 100 μl of TMB substrate solution was pipetted to all wells and the microwell strips were incubated again at room temperature for 10 min. The substrate reaction was stopped by quickly pipetting 100 μl of Stop Solution, and then immediately read for absorbance at 450 nm using a spectro-photometer (SpectraMax Paradigm Multi-Mode Microplate Reader, SoftMax Pro Software, 2014). A standard curve was created by plotting the mean absorbance for each standard concentration on the ordinate against the measured sCD80 concentration on the abscissa.

Group comparison was performed using standard statistical tests: *t*-test for continuous variables, chi square for categorical values and ANOVA test for comparison of more than 2 continuous variables. Analyzing receiver operating characteristic (ROC curves) was assessed using *pROC*, an open-source package (8).

## Results

Fifty-six febrile children admitted to the hospital were approached for consent. 12 patients were excluded, 11 because of insufficient amount of urine and one patient because of positive urine culture. Finally 44 febrile children age 2 months−17.7 years, (6.6 ± 0.79 years, mean ± S.E.M.) were enrolled and compared with 28 controls age 3 months−16.5 years (4.9 ± 0.72 years, mean ± S.E.M., *p* = 0.12). There were 27 (60%) males in febrile group vs. 21 (75%) ones in the control group. Seventeen patients (39%) in febrile group vs. 3 patients (11%) in the control group had some background illness, 18 (41%) in febrile group vs. 8 (29%) in the control group were previously hospitalized (Table 1). Temperature on admission in febrile group was 38.6 ± 0.9°C, maximal temperature was 39 ± 0.74°C, the fever duration prior to admission was similar for both bacterial and viral groups and averaged 31 ± 4 h (mean ± S.E.M). In the study group febrile disease was of bacterial origin in 52% of cases and of viral origin in 48%. Bacterial diagnoses included: pneumonia (9), rickettsiosis (2), dysentery (4), cellulitis with abscess (1), mastoiditis (1), acute otitis media (1), and occult bacteremia (1). The diagnosis of pneumonia was based on positive findings on chest X-Ray examinations (lobar infiltrate). In one patient blood serologic test was positive for Mycoplasma Pneumonia. Blood cultures were negative in all the patients with pneumonia except one which was positive for Pneumococcus Pneumonia. All patients with pneumonia except two had elevated leucocyte count (24 ± 2.6^*^10^3^/ul, mean ± S.E.M.). In the patient with rickettsiosis, the diagnosis was based on clinical and laboratory findings and confirmed serologically. Mastoiditis was diagnosed based on clinical findings and leukocytosis (17^*^10^3^/μl). In the patient with dysentery, stool cultures were positive for shigella (2), salmonella (1), and campylobacter (1). In the patient with an abscess and cellulitis, positive culture for Staph aureus was obtained from the pus. Otitis media was diagnosed clinically, but this patient was also diagnosed with pneumonia, confirmed by a chest X-ray. The diagnosis of occult bacteremia was made based on fever and leukocytosis (20^*^10^3^/μl). Febrile patients without specific focus of infection and normal peripheral blood leucocyte count were diagnosed as having viral infections.

**Table 1:** Demographic characteristics.

	**Febrile**	**Afebrile**	***P* value**
Number	44	28	
Age (mean ± S.E.M.)	6.6 ± 0.79	4.9 ± 0.72	0.12
Male (%)	27 (60%)	21 (75%)	NS
Background illness	17 (39%)	3 (11%)	0.02
Previous hospitalization	18 (41%)	8 (29%)	NS
Temperature on admission (°C), (mean ± S.E.M)	38.6 ± 0.9		
Maximal temperature (°C), (mean ± S.E.M)	39 ± 0.74		
Hours of fever prior to admission (mean ± S.E.M)	31 ± 4		
Suspected bacterial disease (%)	23 (52%)		

Urine samples were obtained 31 ± 4.1 h (mean ± S.E.M.) from the beginning of fever. The assessment of different urinary proteins is summarized in Table 2. Multistix® urinalysis strips were positive for protein (in those samples with specific gravity of more than 1010) in 13 out of 43 febrile patients (30%) and only in 1 patient from the control group (3.7%) (two-tailed χ^2^ = 0.005). In the febrile group urine protein/creatinine ratio was above normal (200 mg/g) in 32 cases (72.2%) vs. 5 cases (18%) in the control group (χ^2^ = 0.001). In the febrile group 27 patients (60%) had a urine albumin\creatinine ratio above 30 mg/g (upper limit of norm) vs. 3 patients in the control group (χ^2^ < 0.005). Urinary albumin\creatinine ratio in the febrile group was 64.5 ± 10.3 mg/g, mean ± S.E.M. Of note, the albumin fraction in urine was less than half of the total protein. There was a wide distribution in urine β2-microglobulin excretion in the study group: 1.44 ± 0.34 mg\g, mean ± S.E.M. In the control group, urine β2-microglobulin was significantly lower (0.182 ± 0.03 mg/g, mean ± S.E.M, *p* = 0.01) (Table 2). Tamm-Horsfall Protein (THP) excretion in urine was significantly decreased in febrile children vs. control (10.5 ± 1 vs. 26.7 ± 2.2 arbitrary units, mean ± S.E.M, *p* = 0.0001).

**Table 2:** Characteristics of proteinuria.

**Urine values (mean ± S.E.M)**	**Normal value**	**Afebrile (*n* = 28)**	**Febrile**		***P*-value AF vs. F**	***P*-value Bacterial vs. Viral**
Protein/creatinine (mg/g)	<200	150 ± 17	Total (*n* = 44)	430 ± 54.5	0.0001	
			*Bacterial* (*n* = 23)	480 ± 83.3		NS
			*Viral* (*n* = 21)	380 ± 65.3		
Albumin/creatinine (mg/g)	<30	17.8 ± 4	Total (*n* = 44)	64.5 ± 10.3	0.0009	
			*Bacterial* (*n* = 23)	44.7 ± 8.85		NS
			*Viral* (*n* = 21)	117.9 ± 33.2		
β2mgl/creatinine (mg/g)	≤0.132	0.182 ± 0.03	Total (*n* = 44)	1.44 ± 0.34	0.005	
			*Bacterial* (*n* = 23)	1.218 ± 0.35		NS
			*Viral* (*n* = 21)	1.693 ± 0.58		
B7-1 (ng/ml)	NA	0.07 ± 0.01	Total (*n* = 25)	0.27 ± 0.05	0.001	
			*Bacterial* (*n* = 11)	0.4 ± 0.07		0.002
			*Viral* (*n* = 14)	0.14 ± 0.04		
Tamm-Horsfall protein (AU)	NA	26.7 ± 2.2	Total (*n* = 44)	10.5 ± 1	0.0001	
			*Bacterial* (*n* = 23)	7 ± 1		0.14
			*Viral* (*n* = 21)	9.5 ± 1.3		

*AF-afebrile, F-febrile, NA-not available, NS-not significant, AU-arbitrary units. AF-afebrile, AU-arbitrary units, β2mgl-beta-2 microglobulin, F-febrile, NA-not available, NS-not significant*.

**Figure 1:**
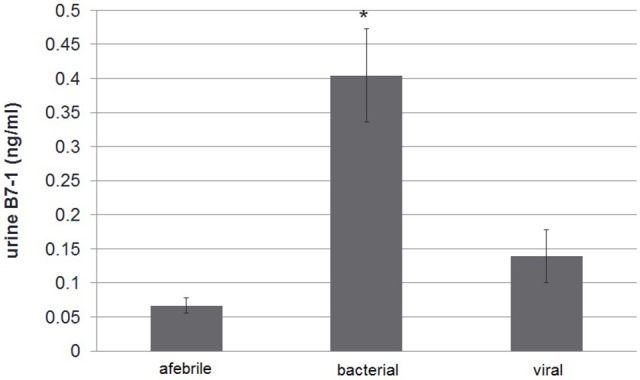
Urine B7-1 excretion in febrile disease of bacterial vs. viral origin and vs. control afebrile group: urine B7-1 concentration was significantly higher in cases of bacterial disease vs. viral and in comparison with the control group *p < 0.0001.

**Figure 2:**
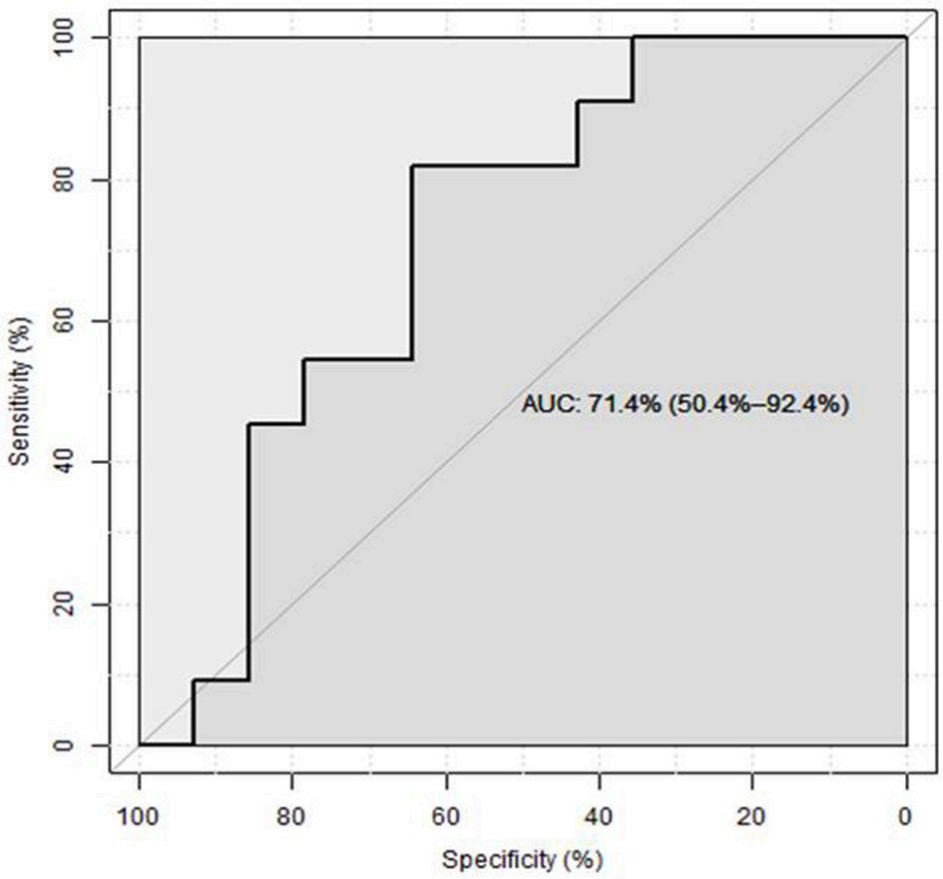
ROC curve assessing the predictive value of urine B7-1 for bacterial infection in febrile children (8).

The study group comparison was further subdivided into three groups: afebrile children, patients having febrile disease of bacterial origin (according to above mentioned criteria) vs. non-bacterial origin. As mentioned, total protein excretion was higher in febrile children vs. controls. However, there was no difference between bacterial and nonbacterial febrile groups. Albumin excretion was also elevated in febrile children vs. control without a clear difference between nonbacterial and bacterial groups (Table 2). There was no significant difference in urine β2-microglobulin excretion between bacterial and nonbacterial groups but its excretion was significantly elevated in each of these groups vs. control (*p* < 0.05). Uromodulin (THP) excretion was lower in the bacterial vs. nonbacterial group, but this difference was not significant (*p* = 0.14). There was a significantly higher excretion of B7-1 in urine in the febrile group vs. afebrile controls (0.27 ± 0.05 vs. 0.07 ± 0.01 ng/ml, mean ± S.E.M., *p* = 0.0001). Urine B7-1 concentration was also significantly higher in cases of bacterial disease vs. nonbacterial (0.4 ± 0.07 vs. 0.14 ± 0.04 ng/ml, mean ± S.E.M., *p* < 0.002) (Table 2) (Figure 1). An ROC curve that tests the performance of urine B7-1 to differentiate between viral and bacterial infections yielded an AUC value (±S.E.M) of 0.85 ± 0.08 (95% CI: 0.71–0.99, *p* = 0.003) (Figure 2). The optimal mathematical point along the curve is a urine B7-1 value of 0.132, providing a sensitivity of 91.7% and a specificity of 61.5%. Thus, there was no significant difference in urine excretion of any of the checked proteins in the cases of bacterial vs. viral infection except for urine B7-1 that was significantly higher in the cases of bacterial infections.

## Discussion

In this study we first characterized the type of urinary proteins in children with febrile proteinuria. Elevated urinary total protein (in the mild-non-nephrotic range), urine albumin and β2-microglobulin excretion were found in febrile children. Urine B7-1 excretion also was higher in the cases of febrile states. Not all urinary proteins showed a pattern of transient elevation during febrile illness. For example, uromodulin (Tamm-Horsfall protein), that is excreted in urine in normal states, was actually significantly decreased during fever. This protein is secreted by the kidney and is thought to play role in water\electrolyte balance and kidney innate immunity. Studies in *Umod* knockout mice showed that uromodulin has a defensive role against urinary tract infection (UTI) (10, 11), since this protein binds to pathogens of the urinary tract, such as type 1-fimbriated *E. coli*, and interferes with their binding to uroplakins on the urothelium (12). Several *in vitro* studies showed that THP is able to bind to immunity-related molecules, such as immunoglobulin G, complement 1q, and tumor necrosis factor-α (9, 13, 14). Uromodulin also acts as a chemoattractant (15) and as a proinflammatory molecule. It interacts with monocytes, neutrophils and myeloid dendritic cells leading to activation of components of the immune system via toll-like receptor 4 (16). The physiological role of this process is still unclear. Saemann et al. hypothesize that uromodulin is released into kidney parenchyma in cases of tubular damage and serves as a signal to activate local immune response to prevent bacterial invasion (16). This unexpected decreased urinary THP excretion during fever can be explained by its excessive excretion into blood, as Prajcer et al. showed that inflammatory damage in thick ascending limb leads to decreased urinary and increased blood uromodulin level (17). Medullary cystic kidney disease type 2 (MIM 603860) and familial juvenile hyperuricemic nephropathy (MIM 162000) are autosomal dominant tubulointerstitial kidney diseases that are due to UMOD gene mutations and are collectively called uromodulin-associated kidney disease (UAKD). Decreased urine uromodulin excretion in these diseases leads to nephropathy, interstitial nephritis, hyperuricemia, renal stone formation and renal insufficiency. In this study, in spite of elevated albumin and β2-microglobulin urine excretion during febrile disease, most of urine protein was neither albumin nor β2-microglobulin. The increase in urinary albumin excretion suggests that additional high molecular weight proteins may also be excreted, most probably due to a transient disturbance in glomerular barrier. Recent studies have reported new evidences on the pathophysiology of proteinuria of glomerular origin, including the role of B7-1. This protein is expressed in glomerular epithelial cells (podocytes) and influences glomerular anatomical barrier in different proteinuric states. B7-1 influences the actin cytoskeleton of podocytes and slit diaphragm organization leading to proteinuria. Both genetic aberrations (e.g., deletion of α3 integrin or nephrin), toxic stimuli (such as PAN induced reactive oxygen species), or direct stimulation of the TLR-4/CD14 receptor on the podocyte can cause B7-1 induction. B7-1 then induces the podocyte's foot process effacement and disruption of the slit diaphragm complex, leading to proteinuria. Reiser et al. (6) also showed rapid upregulation of B7-1 in podocytes and nephrotic-range proteinuria by *in vivo* exposure to low-dose LPS in wild type and SCID mice. B7-1 knockout mice were protected from this LPS induced nephrotic range proteinuria, suggesting a role of podocyte (and not white blood cell) B7-1 expression in the pathogenesis of proteinuria. Podocyte's B7-1 is upregulated in patients with certain glomerular diseases. Positive B7-1 immunostaining was observed in biopsy specimen from patients with recurrent focal segmental glomerulosclerosis (FSGS) a disease associated with severe proteinuria. Yu et al. showed the resolution of nephrotic range proteinuria after B7-1 inhibition with Abatacept (CTLA-4–Ig) treatment in patients with rituximab-resistant recurrent FSGS and in patients with glucocorticoid-resistant primary FSGS (18), suggesting that B7-1 can be a target for the treatment of proteinuria. However, these findings have recently been challenged by Baye et al. (19). These new observations led us to explore the possible role of B7-1 in febrile children. As mentioned, B7-1 was found to be the only urine biomarker that was not just significantly higher in febrile disease vs. controls, but also higher in febrile cases of bacterial disease vs. viral origin.

Activation of toll-like receptors (TLRs) is basic in the initiation of innate immunologic response (20,21). For example, exposure to lipopolysaccharide (LPS) induces B7-1 expression via TLR-4 in podocytes, leading to reorganization of its cytoskeleton, foot process effacement and proteinuria. B7-1 acts as a costimulatory molecule in this process, as previously mentioned (6). We suppose that in the cases of bacterial infections toxins or other bacterial components lead to proteinuria via similar mechanism and B7-1 acts as a co-stimulatory molecule in this process, being upregulated and eventually found in urine.

## Limitations of the study

The study was performed on a relatively small sample of patients, due to budget limitations. Thus, the negative statistically significant differences found between the bacterial and viral groups may become significant in larger study groups. In addition, only a limited number of urinary excreted proteins were analyzed in this study, and the correlation between blood level of these proteins and their excretion was not assessed.

In summary, in this first study we have shown that febrile proteinuria is not a generalized nonspecific phenomenon: while proteins of both glomerular and tubular origin increase, uromodulin decreases. Urine B7-1, a possible marker of increased glomerular permselectivity is increased during fever, more significantly in bacterial infections. Thus, if verified by larger studies, urinary B7-1 may be used as an additional marker to differentiate between febrile states of bacterial and viral origin.

## Ethics statement

The research protocol of the study has been approved by the Soroka University Medical Center local Helsinki committee. Study consent was signed by patients' parents.

## Author contributions

EG and EI recruited the patients. EI wrote a preliminary report. EG wrote the manuscript's first draft. YS performed the laboratory analyses and reviewed the manuscript. DL conceived the study design and finalized the manuscript's last version.

### Conflict of interest statement

The authors declare that the research was conducted in the absence of any commercial or financial relationships that could be construed as a potential conflict of interest.
